# Clinical use of submaximal treadmill exercise testing and assessments of cardiac biomarkers NT-proBNP and cTnI in dogs with presymptomatic mitral regurgitation

**DOI:** 10.1371/journal.pone.0199023

**Published:** 2018-06-14

**Authors:** Leona Wall, Annika Mohr, Florenza Lüder Ripoli, Nayeli Schulze, Camila Duarte Penter, StephanOscar Hungerbuehler, Jan-Peter Bach, Karin Lucas, Ingo Nolte

**Affiliations:** Small Animal Clinic, University of Veterinary Medicine Hannover Foundation, Hannover, Germany; Faculty of Animal Sciences and Food Engineering, University of São Paulo, BRAZIL

## Abstract

Exercise intolerance is the first symptom of heart disease. Yet an objective and standardised method in canine cardiology to assess exercise capacity in a clinical setting is lacking. In contrast, exercise testing is a powerful diagnostic tool in humans, providing valuable information on prognosis and impact of therapeutic intervention. To investigate whether an exercise test reveals differences between dogs with early stage mitral regurgitation (MR) and dogs without cardiac disease, 12 healthy beagles (healthy group, HG) and 12 dogs with presymptomatic MR (CHIEF B1 / B2, patient group, PG) underwent a six-stage submaximal exercise test (ET) on a motorised treadmill. They trotted in their individual comfort speed for three minutes per stage, first without incline, afterwards increasing it by 4% for every subsequent stage. Blood samples were taken at rest and during two 3-minute breaks in the course of the test. Further samples were taken after the completion of the exercise test and again after a 3-hour recovery period. Measured parameters included heart rate, lactate and the cardiac biomarkers N-terminal pro-B-Type natriuretic peptide and cardiac Troponin I. The test was performed again under the same conditions in the same dogs three weeks after the first trial to evaluate individual repeatability. Cardiac biomarkers increased significantly in both HG and PG in the course of the test. The increase was more pronounced in CHIEF B1 / B2 dogs than in the HG. N-terminal pro-B-Type natriuretic peptide increased from 435 ± 195 to 523 ± 239 pmol/L (HG) and from 690 to 815 pmol/L (PG). cTnI increased from 0.020 to 0.024 ng/mL (HG) and from 0.06 to 0.08 ng/ml (PG). The present study provides a method to assess exercise-induced changes in cardiac biomarkers under clinical conditions. The increase of NT-proBNP and cTnI is more pronounced in dogs with early-stage MR than in healthy dogs. Results indicate that measuring the parameters before and after exercise is adequate and taking blood samples between the different stages of the ET does not provide additional information. Also, stress echocardiography was inconclusive. It can be concluded that exercise testing, especially in combination with measuring cardiac biomarkers, could be a helpful diagnostic tool in canine cardiology.

## Introduction

Exercise intolerance as the first symptom of mitral regurgitation (MR) in dogs evolves slowly and progressively and therefore often stays unnoticed until symptoms like coughing occur during exercise [1]. In order to detect these changes as early as possible, submaximal exercise tests (ET) are commonly used in human cardiac patients for the assessment of functional capacity, prognosis, risk stratification and treatment options and for follow up-examinations in patients with heart diseases [2, 3]. Especially in unclear cases, the decision for or against therapeutic intervention is based on results of exercise tests and a post-exercise increase of cardiac biomarkers (CB) is of marked importance for the prognosis [4–7].

Although potentially providing plenty of information, ET are not used routinely in clinical cardiology in dogs. The main reasons for this are the absence of a standardised objective testing method and the fact that dogs vary immensely in regard to size and breed. However, submaximal ET have been used in studies in dogs. Considerable limitations for clinical use have been the implementation of invasive measurement methods [8–10] or the use of an unsuitable treadmill for dogs [11]. A study in canine heart patients lacked a healthy control group and therefore standardisation [12]. For these reasons an objective functional classification of canine heart patients by ET for clinical use is still lacking [1]. This might be why, until now, no study has investigated if exercise tolerance in canine presymptomatic heart disease provides additional information on the prognosis as it does in human patients and could therefore help distinguishing preclinical dogs with MR that might benefit from early medication.

Healthy dogs use their functional reserves in response to exercise by increasing sympathetic activity in combination with a vagotonic decrease. This leads to an increase in cardiac output, which is predominantly dependent on heart rate (HR) [13, 14]. Being chronically activated in heart failure patients, these mechanisms do not function as effective as in healthy dogs, leading to worse adjustment to exercise and thus exercise intolerance [15]. This autonomic balance can be (partially) restored by exercise training as an adjuvant therapy [16, 17]. In human patients, stress echocardiography has been shown to be helpful in clinical decision-making [2]. Parameters include cardiac output [2] and also pulmonary artery pressure as an indicator of dynamic changes in MR [18].

The cardiac biomarkers NT-proBNP and cardiac troponin I (cTnI) are promising parameters for canine cardiology, yet they are mostly used for research purposes, as solid characterisations for the use in a clinical setting are lacking [19, 20]. Resting values are subject to day-to-day variability and are influenced by age or breed and circadian rhythms [21–24].

NT-proBNP and its mature form B-type natriuretic Peptide (BNP) are markers of cardiac wall stress [25]. They can serve as a helpful tool in differentiating healthy dogs from dogs with congestive heart failure [25–27] or congenital heart anomalies [28]. Studies in humans with asymptomatic MR emphasised the importance of exercise-induced increases of BNP, as patients with an elevated exercise BNP were at high risk to experience a cardiac event [5, 7]. Previously it has been shown that the length of the regurgitant jet is less important than the hemodynamic consequences [29]. As these can differ during exercise and rest [4], findings from echocardiography at rest might be supplemented by post-exercise NT-proBNP values. Furthermore, exercise training leads to a decrease of baseline NT-proBNP values in dogs with MR of different stages [17].

Usually indicating myocardial damage [30], cTnI also increases after endurance exercise [31–33] and does so even more, when dogs with heart failure undergo ET [34]. In addition to this, a study in humans found a higher increase of cTnI after exercise in patients with moderate heart failure compared to healthy individuals and patients with mild heart failure [6]. Reason for elevated post-exercise cTnI levels are leakages in the cell membrane of the cardiomyocytes due to strain because of an increased cardiac output [35, 36].

As patient classification in canine cardiology is based solely on results of clinical examinations at rest, it remains unknown whether ET in dogs with presymptomatic MR is suitable to detect early onset of exercise intolerance and might therefore provide additional information on the patient’s current status and the prognosis. Thus, the purpose of the present study is to investigate if results of a submaximal ET in dogs with presymptomatic MR differ from a healthy control group with particular focus on the exercise-induced changes in the cardiac biomarkers cTnI and NT-proBNP.

## Materials and methods

This study was approved by an institutional ethics commission (Lower Saxony State Office for Consumer Protection and Food Safety; 33.9-42502-05-14A484).

### Animal selection

All dogs underwent physical examination, blood count and serum biochemical analysis, urinalysis, electrocardiogram and echocardiography. The dogs were included if there was no evidence of a systemic or orthopaedic disease which could affect exercise performance on the treadmill or influence biomarker concentration (e.g. lameness, renal or respiratory diseases). Further inclusion criteria were absence of any history of cardiac symptoms or medication.

Twelve healthy beagle dogs aged 3 ± 2 (mean ± SD) years served as a healthy control group (HG). The dogs weighed 13 ± 3 kg and had a body condition score of 4–7. The group consisted of 3 neutered females, 5 intact and 4 neutered males. The Beagles were obtained from university institutes (and University of Veterinary Medicine, Hannover, foundation, Hannover, Germany) or from a breeder for use in research (Envigo, formerly Harlan Laboratories, Gannat, France). The dogs were raised in the clinic for small animals at the University of Veterinary Medicine, Hannover, foundation. They lived in pens in groups of a maximum of 6 dogs with daily access to a separate open-air area. They were fed commercial dry food for adult dogs twice daily according to the manufacturer’s instructions and had unlimited access to water.

Twelve dogs of various breeds with a typical heart murmur of at least grade 1/6 over the mitral valve area (patient group, PG) were also enrolled in this study (Table 1). Owners had given informed consent to participate in the study. The dogs were 9.5 ± 3 years of age (significantly older than the HG), weighed 18 ± 9 kg and had a body condition score of 4–7.

**Table 1:** Breed, age, sex and weight of dogs enrolled in the patient group.

Patient no.	Breed	Age (years)	Sex	Weight (kg)
1*	Beagle	8	SF	19
2 #	Beagle	12	F	14.8
3*	Beagle	8	NM	13.6
4*	Beagle	5	NM	18.3
5*	Mixed-breed	6	SF	7.6
6*	West Highland White Terrier	11	F	9.0
7*	Weimaraner	10	NM	32.2
8*	Jack-Russel	12	SF	8.7
9*	Mixed-breed	15	M	11.5
10#	Galgo Español	9	SF	19.1
11*	Golden Retriever	8	M	28.7
12*	Golden Retriever	8	SF	34.5

*CHIEF B1,

^#^ CHIEF B2,

F: female, M: male, NM: neutered male, SF: spayed female

### Echocardiographic measurements

Every dog was examined by an experienced cardiologic investigator using an ultrasound system (Vivid E9, GE Healthcare, Solingen / Germany; right and left lateral recumbency, 2-dimensional, M-mode and Doppler examination).

The HG and 3 dogs of the PG additionally underwent stress echocardiography. They were positioned in right lateral recumbency for image acquisition. Stroke volume was calculated according to Teichholz and the Simpson method of disks [37, 38]. Further parameters included maximal aortic blood flow velocity and blood flow in the pulmonary artery (ratio of the acceleration time to ejection time). All echocardiographic examinations were performed directly before and after exercise by the same investigator (S. O. Hungerbuehler). All values were measured 3 times and were averaged.

### Treadmill test

The exercise test consisted of a 6-stage protocol on a motorised treadmill (“quasar”, h/p/cosmos sports and medical GmbH, Nussdorf-Traunstein / Germany) with a duration of three minutes per stage, starting with 0% incline, increasing by 4% at each stage. The stages and measurements are summarised in Table 2.

**Table 2:** Stages of the exercise test and measurements. Time points at which measurements were performed are marked with an “X”.

Stage (incline) / Parameter	Prior to exercise	1 (0%)	2# (4%)	3 (8%)	4# (12%)	5 (16%)	6 (20%)	Recovery
NT-proBNP	X						X	
cTnI	X							X
Heart Rate	X	X	X	X	X	X	X	X
Lactate	X		X		X		X	X
Bicarbonate	X		X		X		X	X
pH value	X		X		X		X	X

X: the measurement was performed at that time point

^#^: blood samples were collected during a 3-minute break at the end of the stage

NT-proBNP: N-terminal pro-B-Type natriuretic peptide. cTnI: cardiac troponin I

Each dog trotted at its individual comfort speed, which was defined as the speed at which the dogs trotted freely in a steady and clear 2-beat movement without trying to brake or accelerate for at least 30 seconds. This speed was used in all treadmill examinations. To ensure a minimal workload, heart rate (HR) at stage 2 had to exceed 150 beats per minute (bpm) or be raised by 40% compared to values obtained at rest. To ensure that the exercise remained submaximal, the maximal HR was 240 bpm [2, 9]. In case of an individual’s HR exceeding this, the ET would have been cancelled. During the exercise protocol, each dog received a maximum of 50 g of food for motivation.

The dogs were familiarised with the treadmill prior to the first trial. The duration of each session was less than 10 minutes including multiple breaks. All dogs rested for at least 15 minutes before venous blood samples for baseline measurements were taken.

Room temperature was kept between 21 and 23 °C during all ETs to ensure the same ambient conditions. Each dog from the HG and 9 dogs from the PG underwent the ET with the same individual speed twice at an interval of 3 weeks to verify the repeatability of the measured parameters. Due to various outliers in the first trial, one dog of the HG was retested 8 weeks after the second trial and the first trial’s results were discarded. Another dog of the HG was playing intensively with other dogs in the pen shortly before samples were taken at recovery, so the data for that time point were also excluded from analysis for that day.

### Measurements

Blood was collected from the vena saphena or vena cephalica antebrachii. RF was determined by counting the number of chest rises at rest, after stages 2, 4, after the end of the ET and at recovery. In case of panting, values were not recorded. HR was measured constantly by a Polar^®^ heart rate monitor with an electrode belt (Polar FT7N and Polar H1, Polar Electro GmbH Deutschland). It was obtained by averaging 5 consecutive values that were recorded during the last 30 seconds of each stage of exercise (during trotting) and at rest directly before the ET.

Blood samples for oxygen and carbon dioxide partial pressure (PO_2_ and PCO_2_) analysis and acid-base status (pH and bicarbonate) were taken anaerobically and stored on ice until analysis took place within a 10 minute timespan (Rapidlab 1260, Siemens Healthcare Diagnostics GmbH, Eschborn / Germany). Samples for lactate assessment were inserted into tubes containing fluorid heparine and measured immediately in a clinical chemistry analyser (Cobas c 311 Analyser, Roche Diagnostics, Mannheim / Germany).

Because cTnI has been reported to reach peak serum levels hours after a cardiac event [34, 39], it was measured before and 3 hours after the ET. Samples for cTnI and NT-proBNP were stored on ice within seconds after extraction. After subsequent centrifugation within a 10 minute timespan, the serum (cTnI) or EDTA plasma (NT-proBNP) respectively was frozen at -20 °C before being transferred into a -80 °C freezer until shipment on dry ice to the laboratory (IDEXX Laboratories, Ludwigsburg / Germany) took place. cTnI was measured by an ultra sensitive chemiluminescence assay and NT-proBNP by ELISA.

### Statistical procedures

The number of animals to be used was calculated by a power analysis based on the following assumptions: likelihood of type I error: 0.05, likelihood of type II error: 0.2, biologically relevant difference in cTnI: 0.15, variance of effect size 0.01, power 0.8.

The data of cancelled ETs (n = 2 each in the HG and PG) were excluded from analysis for the time points after termination (except for cTnI and NT-proBNP). The data of the first trial are presented as mean ± standard deviation. The data or their residuals, respectively, were tested for normal distribution by a Shapiro-Wilk test.

The values obtained on the first and second trial were compared using the intraclass correlation coefficient (ICC) with a 95% confidence interval (mixed model, absolute agreement). An ICC > 0.7 indicated repeatability of the data [40].

Differences in the measured parameters were analysed using a one-way repeated measures analysis of variance (ANOVA) with Tukey’s honestly significant difference post hoc test. For parameters with only 2 time points (NT-proBNP and cTnI), a paired t-test was used. Values from the HG were compared with the values from the PG using a 2-sample t-test. Results were regarded as significant, if the P-value was ≤ 0.05.

## Results

The submaximal ET was easy to perform and every dog was willing to run on the treadmill. All 24 dogs underwent the ET at a speed of 4.2 to 8 km/h and none of the ET had to be terminated due to an excessive increase of HR. Each dog showed increases of HR above 150 bpm or raised their resting HR by at least 40% at stage 2, indicating that the criteria for a minimum workload were met. One dog from the HG and 2 dogs from the PG refused to exercise further than stage 5 in both trials. With another dog from the HG, the test had to be stopped due to technical difficulties at stage 5 in the first trial.

Significant differences between healthy and CHIEF B1 / B2 dogs were apparent in CB and in some other parameters (see below) but not at every level of exercise.

In Table 3 the ICC values are listed. Although the ICC did not exceed 0.7 at every time point, significant differences between distinct time points were found on both trials. The ET yielded workload-dependent alterations in the parameters NT-proBNP, cTnI, HR, lactate, bicarbonate and pH.

**Table 3:** Intraclass correlation coefficient of the variables between the first and the second trial at different exercise stages during graded treadmill exercise.

Stage / Parameter		Prior to exercise	Stage 2	Stage 4	After exercise	Recovery
NT-proBNP	HG	**0.85**	-	-	**0.90**	-
	PG	**0.92**	-	-	**0.95**	-
cTnI	HG	0.55	-	-	-	0.60
	PG	**0.99**	-	-	-	**0.94**
Lactate	HG	<0.50	0.54	**0.75**	<0.50	<0.50
	PG	**0.89**	**0.82**	**0.89**	**0.96**	**0.79**
Bicabonate	HG	<0.50	0.59	**0.72**	0.63	<0.50
	PG	**0.8**	0.65	**0.78**	<0.50	<0.50
pH	HG	<0.50	<0.50	<0.50	**0.77**	<0.50
	PG	<0.50	**0.84**	**0.9**	**0.92**	**0.89**

- indicates the value was not measured at that stage; values ≥ 0.7 are highlighted in bold

HG: Healthy group, PG: Patient group, pH: potential of hydrogen, cTnI: cardiac troponin I; NT-proBNP: N-terminal pro B-type natriuretic peptide

### Cardiac biomarkers

NT-proBNP increased by 124 pmol/l (P ≤ 0.01) after exercise in the PG (Table 4), which was more pronounced than in the HG (increase by 88 pmol/l), but not statistically significant. Baseline values for NT-proBNP were significantly higher in PG than in HG (690 vs. 435 pmol/l, P ≤ 0.05).

**Table 4:** Means and SD of the variables at different stages during graded treadmill exercise in healthy and CHIEF B1 / B2 dogs.

Parameter	Group	Prior to exercise	Stage 2	Stage 4	After exercise	Recovery
NT-proBNP (pmol/l)	HG	*435 ± 195	-	-	523 ± 239	-
	PG	*690 ± 345	-	-	815 ± 417	-
cTnI (ng/ml)	HG	*0.02 ± 0.01	-	-	-	*0.024 ± 0.01
	PG	*0.06 ± 0.06	-	-	-	*0.08 ± 0.06
Lactate (mg/dl)	HG	9.78 ± 2.6	8.3 ± 2.3	10.13 ± 4.44	11.5 ± 3.5 (n = 10)	*9.5 ± 1.8 (n = 10)
	PG	12.2 ± 4.7	9.8 ± 4.8	14.0 ± 6.7	14.43 ± 9.8 (n = 10)	*14.33 ± 4.9 (n = 10)
Bicarbonate (mmol/l)	HG	*21.0 ± 1.3	22.2 ± 2.2	21. ± 1.9	*19.7 ± 1.8 (n = 10)	22.3 ± 2.0 (n = 10)
	PG	*22.7 ±1.8	23.29 ± 2.3	21.9 ± 1.5	*20.7 ± 1.2 (n = 10)	22.7 ± (n = 10)
pH	HG	7.40 ± 0.02	7.40 ± 0.03	7.41 ± 0.03	7.47 ± 0.05 (n = 10)	7.37 ± 0.03 (n = 10)
	PG	7.41 ± 0.04	7.42 ± 0.05	7.43 ± 0.04	7.46 ± 0.03 (n = 10)	7.39 ± 0.04 (n = 10)

pH: potential of hydrogen; cTnI: cardiac troponin I; HG: healthy group (n = 12); NT-proBNP: N-terminal pro B-type natriuretic peptide; PG: patient group (n = 12)

* indicates a significant difference between HG and PG at that stage, n is indicated in brackets if there were less than 12 dogs from which samples were available at that stage

cTnI increased by 0.004 ng/ml (HG, P ≤ 0.05) and 0.02 ng/ml (PG, P ≤ 0.001). Values were higher in PG than HG at rest (0.06 vs 0.02 ng/ml, P ≤ 0.05) and at recovery (0.08 vs 0.02 ng/ml, P ≤ 0.05) and the increase of cTnI was more pronounced in the PG (P ≤ 0.05).

Pre and post-exercise values of NT-proBNP showed excellent repeatability in both groups. cTnI in the PG also yielded a high ICC. Also the quotient of recovery to resting values of cTnI in the HG was 0.69 when all the data were analysed and 0.8 when data from the 2 dogs that did not undergo the whole ET were excluded.

### Heart rate

HR was significantly (p ≤ 0.001) higher at any level of exercise than at rest (Table 5) and yielded predominantly repeatable results. Mean HR did not differ between the HG and PG at the different levels of exercise and also relative increase of HR to resting values did not differ between the groups.

**Table 5:** Heart rate (mean ± SD) on different levels of exercise.

Group / Stage	Prior to exercise	Stage 1	Stage 2	Stage 3	Stage 4	Stage 5	Stage 6
HG	119 ± 27	166 ± 19	169 ± 21	179 ± 17	182 ± 29	187 ± 19	192 ± 26
PG	117 ± 26	166 ± 18	168 ± 21	171 ± 20	181 ± 22	186 ± 20	190 ± 26

HG: Healthy group, PG: Patient group

### Lactate

Initially lactate decreased but further workload led to an increase (Table 4). After the 3-hour recovery period it was significantly higher in the PG than HG (14.33 ± 4.9 vs 9.5 ± 1.8 mg/dl, P ≤ 0.05) but was not significantly different during the exercise protocol.

### Acid-base-status

Values for pH barely changed during the ET (Table 4) but showed a significant (P <0.001) difference between resting values and values after exercise. No significant difference between HG and PG was found.

Bicarbonate showed an initial increase and then decresed with further workload. It was significantly higher in PG than in HG before (22.7 vs. 21.0 mmol/l) and after exercise (22.7 vs. 22.3 mmol/l).

### Respiratory frequency and blood gases

Due to panting in response to exercise, the respiratory frequency did not reveal any relevant information. Blood gases were measured from venous blood samples, therefore showed a great variation.

### Echocardiography

The HG showed no evidence of heart disease in the sonographic examination.

MR in the PG was confirmed by colour Doppler echocardiography, indicated by a regurgitant jet during systole at the mitral valve. Two of the 12 dogs in the PG were classified as CHIEF B2 and the remaining 10 dogs were staged CHIEF B1 based on the results of echocardiography [1]. Values of the parameters obtained during stress echocardiography were not repeatable and yielded no significant result, as exercise HR decreased too rapidly while dogs were placed on the examination table. However, faster assessment of the parameters was not possible.

## Discussion

The submaximal ET investigated in the present study introduces a method for assessing the physiological reaction of selected cardiovascular parameters under predefined exercise conditions in healthy dogs and dogs with presymptomatic MR (CHIEF B1 / B2). It yielded workload-dependent alterations in the parameters lactate, bicarbonate, HR, pH, cTnI and NT-proBNP and can be performed in a clinical setting. The most promising results include assessment of cardiac biomarkers.

Measuring the parameters at different stages of exercise revealed that significant changes occur predominantly between resting values and values after the last stage of the ET. They showed better repeatability than those values, which were measured during the exercise protocol. Therefore it can be concluded, that it is reasonable to abandon blood sample collection between stages and instead measure blood parameters before and after exercise only. However, it is important to observe HR continuously to register values above a submaximal level and ensure a minimal workload.

Although CB concentration can be influenced by age, this can be due to decreased renal function [41] that might occur in older individuals [42]. In order to ensure renal health in the study population, biochemical analysis and urinalysis were performed in every dog and all measurements were within the reference range. Serum BNP concentration did not correlate with age in German Shepherd dogs [43] and a study in humans found neither age nor body mass index to be correlated with exercise-induced increases in BNP [44]. A recent study showed that cTnI elevates in young people after (half-) marathon running, whereas older individuals did not show alterations [45]. Consequently, the increase in CB that was observed in the present study is more likely due to the heart disease than other differences among study populations. Still, the effect of age should be assessed in further studies and as the HG consisted of younger dogs than the PG, this is a limitation of this study.

NT-proBNP concentrations vary immensely between breeds and also show a great intrabreed variation [19, 23]. The same is not true for cTnI levels [46]. In the present study, whereas the HG consisted of Beagle dogs, there were dogs of different breeds enrolled in the PG, which should be pointed out as another limitation of the study. Due to a small sample size, it was not possible to assess the impact of the breed on the results. Although this should be considered, we expect the strain of the exercise to be the predominant effect on exercise CB levels.

Blood sampling has to be performed twice for assessment of resting and exercise CB, but the results of this study indicate, that the diagnostic benefit of the procedure justifies the additional effort. The baseline measurement can also serve as a control value for future examinations. Testing resting values has become an important aspect of daily clinical cardiology in small animal patients and increased CB levels can indicate a variety of diseases of cardiac- or non-cardiac cause. Still, it has been shown, that resting values of NT-proBNP vary between different breeds and also within an individual [47]. Also, baseline values decrease with initiation of exercise training [16, 17]. For these reasons, assessing post-exercise NT-proBNP could gain similar important diagnostic and prognostic value as in human cardiac patients [5, 7].

To the best of our knowledge, this is the first study that demonstrates the impact of MR on differences in CB serum concentrations after submaximal exercise by directly comparing the post-exercise values of healthy dogs to patients with presymptomatic MR. Significant mild increases of cTnI have already been reported in healthy dogs after sled races [31–33], where workload was a lot higher than in the submaximal exercise protocol. A slightly increased exercise cTnI was found in dogs with heart failure (CHIEF C) that underwent submaximal ET [34]. While samples were drawn 3 h post exercise in the present study, Ferasin et al. (2007) measured cTnI immediately after exercise. However, it has been demonstrated that serum cTnI peaks a few hours after a cardiac event like myocardial damage, exercise etc [39]. This is why NT-proBNP might be a parameter that is more feasible for the ET in a clinical setting.

Results in the PG showed a better repeatability for every parameter than in the HG, which might be due to a decreased metabolic flexibility that has been shown for older men [48], because dogs in the PG were significantly older.

Lactate accumulation tended to be increased in the PG compared to the HG, but a significant difference was only found 3 hours after exercise. A study showed that acute congestive heart failure leads to increased baseline lactate levels in dogs and cats [49]. Clearly, the dogs in the present study were in a preclinical stage of their cardiac disease and besides cardiovascular diseases, aging also lowers cardiac output and maximal oxygen consumption [50, 51]. As muscle capillarity also decreases with age, oxidative capacities might not be as high as in younger individuals, leading to an increased lactate accumulation, which has already been demonstrated in older rats [52].

Performing stress echocardiography was not successful in our study. The main reason for this is most likely the delay between ET and image acquisition, which occurred while the dog was taken from the treadmill to the sonography table and prepared for sonography, because HR decreased rapidly in this time. Other studies implemented a dobutamine infusion for cardiac stress testing [43, 53], but as side effects can occur, this method is not applicable as diagnostic procedure in canine clinical cardiology.

During exercise, the cardiac output is increased predominantly by an increase in HR, which is the only parameter that can be easily monitored telemetrically and indicates the strain of the exercise on the dog [13]. Measurement is therefore mandatory in canine exercise testing, although no differences were found between the groups. So far, an impairment in HR adjustment was found in (human) patients in heart failure [15]. Therefore, a significant difference might be present in dogs in a more advanced stage of heart disease than the PG in the present study. According to exercise tests in humans, 85% of the maximal HR should not be exceeded in submaximal protocols [2], therefore the calculation was based on the findings of different studies, which reported a maximal HR of 280 bpm in dogs [9].

Therapeutic intervention using vasoactive medication in dogs with presymptomatic MR (stage CHIEF B1 / B2) is controversially discussed [54, 55]. A recent study demonstrated beneficial effects of Pimobendan in CHIEF B2 dogs [56] and the preclinical phase in dilated cardiomyopathy was prolonged in Dobermann Pinschers [57]. This suggests that early intervention can prolong the presymptomatic phase, but so far it is not possible to predict the timespan of the onset of heart failure. It might be a promising approach to identify individuals among dogs with presymptomatic MR that already show exercise intolerance by ET or elevated exercise CB concentrations, as suggested in human studies: whereas resting values of NT-proBNP were not suitable to differentiate individuals at higher risk, increased post-exercise NT-proBNP was of prognostic value [5, 7].

Owners see quality of life as an even more important factor than survival time, and therefore, improvement of quality of life is an important goal to be reached by therapeutic intervention [58]. As quality of life is also dependent on exercise performance, an ET could also provide valuable information on how the impact of cardiac medication can be evaluated, for instance if exercise-induced increase of CB can be reduced and if it improves or at least maintains exercise tolerance in dogs with presymptomatic MR (CHIEF B1). Also, it could be used as an improved and objective method to monitor the efficacy of a training program on exercise tolerance, which has been used as an adjunctive therapy to cardiac medication [16, 17].

Measuring the parameters before and after exercise is adequate and blood sampling between the other stages did not provide any additional information in our study. However, continuous HR measurement is obligatory. Parameters, that provide the most promising results include CB. cTnI differentiated better between the HG and PG, but NT-proBNP has been shown to be helpful in human patients in long-term investigations [5, 7], therefore both biomarkers should be measured in further studies.

Limitations of the present study include differences of breed, housing and age between the groups, although the exercise-induced increase of CB might not have been affected by these factors, contrary to the baseline values.

The present study is the first approach to assess several parameters of cardiac health in a submaximal ET on a treadmill comparing healthy and dogs with presymptomatic cardiac disease. It can be easily performed in a clinical setting and can also serve as a basis for further studies, especially for observations of therapeutic interventions. Results showed an exercise-induced, significant increase of CB NT-proBNP and cTnI in dogs with early-stage MR, which is more pronounced than in healthy dogs. Though further studies are necessary to assess the impact of age and breed on the results and also to determine corresponding reference values, the submaximal ET presented in this study seems suitable as a new tool in canine clinical cardiology. It could provide additional value regarding prognosis and therapy as exercise tests have shown to do in human patients [2–4].
